# Comparative Study of the Acromioglenoid Angle and Critical Shoulder Angle in Assessing the Role of Scapular Morphology in Primary Glenohumeral Osteoarthritis

**DOI:** 10.7759/cureus.102537

**Published:** 2026-01-29

**Authors:** Lalit Ratanpara, Abhishek Kumar Mishra, Prateek Sihag, Neha Xalxo, Pradip R Chauhan, Simmi Mehra

**Affiliations:** 1 Anatomy, All India Institute of Medical Sciences, Rajkot, Rajkot, IND; 2 Orthopaedics and Trauma, All India Institute of Medical Sciences, Rajkot, Rajkot, IND; 3 Radiology, All India Institute of Medical Sciences, Rajkot, Rajkot, IND

**Keywords:** acromioglenoid angle, critical shoulder angle, glenohumeral joint osteoarthritis, radiological assessment, scapular morphology

## Abstract

Introduction

Glenohumeral osteoarthritis (GHOA) is a common degenerative shoulder disorder. Individual scapular morphology, including glenoid inclination and acromial extension, has been implicated in shoulder biomechanics and disease risk. This study was conducted to assess the comparative efficacy of acromioglenoid angle (AGA) and critical shoulder angle (CSA) as radiological parameters for predicting GHOA.

Methods

A cross-sectional observational study was conducted at All India Institute of Medical Sciences, Rajkot, involving 277 shoulder radiographs in true anteroposterior (AP) view. Participants were divided into GHOA and non-GHOA groups based on radiographic findings. Both CSA and AGA were measured independently by two observers using standardized methodology. Statistical analysis included the intraclass correlation coefficient (ICC) for reliability, Pearson correlation for association analysis, and logistic regression for predictive value determination.

Results

Among 277 shoulders analysed, 158 showed radiographic evidence of GHOA (mean age 65.3 ± 12.4 years; 91 males, 67 females) and 119 were non-GHOA group (mean age 52.1 ± 10.8 years; 62 males, 57 females). Mean CSA was significantly lower in the GHOA group (32.5° ± 2.8°) compared to the non-OA group (36.9° ± 2.3°; t = 13.95, p < 0.001). Similarly, mean AGA was significantly reduced in GHOA patients (44.4° ± 2.7°) versus the non-OA group (50.1° ± 2.8°; t = 17.1, p < 0.001). Strong positive correlation existed between AGA and CSA in both GHOA (r = 0.530, p < 0.001) and non-GHOA groups (r = 0.703, p < 0.001).

Conclusions

Both AGA and CSA are useful radiological measures for predicting GHOA, and there are statistically significant correlations between them. When measuring from the midglenoid reference point, AGA is a useful addition to CSA for clinical assessment and surgical planning for individuals with GHOA.

## Introduction

Primary glenohumeral joint osteoarthritis (GHOA) is characterized by degenerative changes in articulating surfaces and accounts for an estimated 5-17% with shoulder complaints [1,2]. The etiopathogenesis of shoulder joint osteoarthritis (OA) is complicated and involves multiple factors [3,4]. Conversely, the atraumatic degenerative rotator cuff injury and primary GHOA are less extensively researched and understood conditions. Both hereditary and acquired predisposing factors have been linked to these degenerative shoulder conditions [5,6]. Furthermore, individual anatomical variations of the scapula have been directly associated with the development of degenerative shoulder conditions [7]. This variation pertains to either the acromion or the glenoid and can be evaluated through the application of multiple radiological markers. Variations on the acromial side encompass acromial type, acromial slope, and acromial index, whereas variations on the glenoid side include glenoid inclination and version [8-12].

In 2013, Moor et al. introduced the critical shoulder angle (CSA) as a means to quantify the combined influence of glenoid tilt and acromial length within a single radiographic measure. The authors noted in the same study that a higher incidence of GHOA was observed in subjects with lower CSA values [13].

Viehöfer et al. conducted testing of the aforementioned hypothesis using a shoulder simulator and observed increased joint reaction forces associated with lower CSA values, indicating that reduced CSA may contribute to joint overload and the eventual development of GHOA [14].

Recently, Miswan et al. proposed the acromioglenoid angle (AGA) as an alternative radiological measurement utilizing the mid glenoid point as the reference landmark [15]. The rationale for this reference point stems from biomechanical evidence suggesting that the glenohumeral joint reaction force is centered at the mid glenoid during arm elevation, and the humeral head achieves maximum stability through rotator cuff muscle contraction at this anatomical position [16].

Despite promising initial results, comparative validation of AGA against the established CSA parameter remains limited in the literature. Given the importance of scapular geometry in shoulder biomechanics and the growing interest in radiographic risk factors, we performed a radiological cross-sectional study to examine the validity of AGA, a newly suggested parameter, and to compare both parameters in predicting GHOA. It is hypothesized that both angles will be lower in OA patients, but one may show a stronger statistical relationship with OA development.

## Materials and methods

A cross-sectional, observational type of research study was carried out in the outpatient department of the Department of Orthopaedics and the Department of Anatomy at the All India Institute of Medical Sciences (AIIMS), Rajkot, India. The study duration was two years, following permission from the Institutional Ethics Committee (IEC) of AIIMS, Rajkot. This project was carried out from May 2024 to October 2025. This study was undertaken as an intramural research initiative (non-funded) within the Department of Anatomy, in collaboration with the Departments of Orthopaedics and Radiology at AIIMS, Rajkot. The proposal was submitted and presented to the Research Review Board (RRB), and following its approval, it was forwarded to the IEC of AIIMS, Rajkot. The IEC sanction was granted on March 27, 2024, under protocol ID IM/16/NF/2024-25. The sanction letter bearing reference number O.W.No./AIIMS Rajkot/IEC/35/2024, dated March 27, 2024, has been issued. Following IEC approval, the project was submitted to the Clinical Trials Registry-India (CTRI), and registration was successfully approved on May 6, 2024, under registration number CTRI/2024/05/066789.

A convenient sample of 277 patients who presented with non-traumatic unilateral shoulder pain was included in the study. Inclusion criteria were patients aged ≥ 30 years with available high-quality true anteroposterior (AP) view shoulder radiographs. This age criterion was selected because of the various epiphyses of the scapula fused with bone by about the 20th year of age [17]. Exclusion criteria included a history of shoulder fracture, previous arthroscopic or open shoulder surgery, inflammatory arthritis, and rotator cuff tear to avoid confounding. Patients were screened by history and thorough clinical examination, and were subjected to a standardized true AP view of the affected shoulder joint. This X-ray was done as a routine baseline radiological investigation for presenting symptoms. The standardized AP view was utilized, where the affected shoulder is rotated 30 degrees with the arm in neutral position, the elbow straight, the thumb pointing forward, and the patient’s scapula against a radiographic cassette. As per the method suggested by Nyffeler et al., the humeral head will be placed in a neutral position or rotated 20º internally at maximum [10]. An image with ≤5 mm of overlapping between the anterior and posterior margins of the glenoid will be considered an acceptable quality radiograph [18].

The radiographic images were obtained in DICOM format and transferred to the RadiAnt DICOM viewer (Medixant, Poznań, Poland). All measurements were performed in the RadiAnt DICOM viewer software in 2D images. Narrowing of the joint space, the presence of osteophytes, and subchondral sclerosis were observed for the diagnosis of primary GHOA. The diagnosis of primary GHOA was subjective and done by authors AM and PS. Patients with any discrepancies in diagnosis between the two authors were excluded from the study.

Based on radiographic findings, patients were divided into two groups - first group of patients with primary GHOA evident by X-ray findings, and the second group of patients without any arthritic changes. All the measurements were taken by two independent observers who were blinded to clinical status and group assignment.

**Figure 1:**
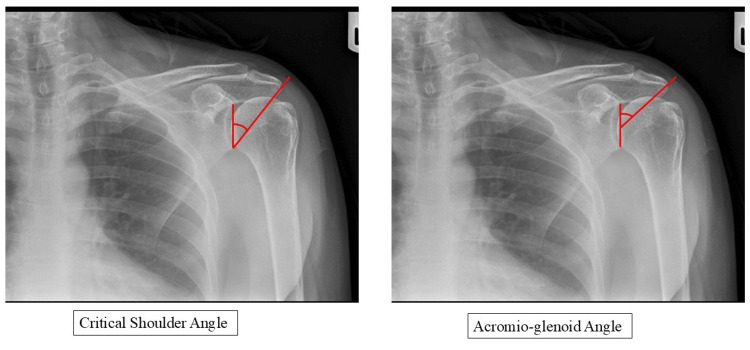
Measurements of CSA and AGA. CSA: critical shoulder angle; AGA: acromioglenoid angle

CSA was the angle subtended, measured by tracing a line from the superior edge of the glenoid to its inferior edge (across the plane of the glenoid fossa), and a second line from the inferior edge of the glenoid to the lateral-most extent of the acromion [13]. AGA was assessed by measuring the angle between a line drawn from the superior edge of the glenoid to its inferior edge and a second line extending from the midpoint of the first line to the lateral-most extent of the acromion (Figure 1) [15].

Both angles were measured using the angle measurement tool integrated within the RadiAnt DICOM viewer. AM and PS performed independent measurements, and LR repeated measurements after a four-week interval to assess intra-observer reliability.

Statistical analyses were performed using IBM SPSS Statistics for Windows, Version 20 (Released 2011; IBM Corp., Armonk, New York, United States). Continuous variables were presented as mean ± standard deviation, and categorical variables were presented as counts and percentages. The strength of association between CSA and AGA was evaluated using the Pearson correlation coefficient (r). A p-value <0.05 was considered statistically significant.

## Results

A total of 277 shoulder radiographs met the inclusion criteria and were included in the analysis. The GHOA group comprised 158 shoulders (91 male, 67 female; mean age 65.3 ± 12.4 years; range 35-87 years), while the non-OA group comprised 119 shoulders (62 male, 57 female; mean age 52.1 ± 10.8 years; range 25-82 years).

A statistically significant difference was observed in both CSA and AGA values between GHOA and non-OA groups. The mean CSA in the GHOA group was 32.5° ± 2.8° compared to 36.9° ± 2.3° in the non-OA group (t = 13.95, p < 0.001), representing a mean difference of 4.4 degrees. Similarly, the mean AGA in the GHOA group was 44.4° ± 2.7° compared to 50.1° ± 2.8° in controls (t = 17.1, p < 0.001), representing a mean difference of 5.7 degrees (Table 1).

**Table 1:** Comparison of radiological parameters and demographic characteristics between GHOA and non-OA groups. Values are expressed as mean ± SD. CSA: critical shoulder angle; AGA: acromioglenoid angle; GHOA: glenohumeral osteoarthritis; OA: osteoarthritis

Parameter	GHOA Group (n = 158)	Non-OA group (n = 119)	t-value	p-value
CSA (degrees)	32.5 ± 2.8	36.9 ± 2.3	13.95	<0.001
AGA (degrees)	44.4 ± 2.7	50.1 ± 2.8	17.1	<0.001
Age (years)	65.3 ± 12.4	52.1 ± 10.8	12.14	<0.001

Progressive age-related decline was observed in both CSA and AGA values across age groups. The youngest cohort (30-39 years) demonstrated the highest mean values (CSA 36.1° ± 2.5°; AGA 49.2° ± 3.2°), while the oldest cohort (≥80 years) showed the lowest values (CSA 31.4° ± 4.9°; AGA 43.3° ± 3.1°). The differences between age groups were statistically significant for both parameters (Table 2).

**Table 2:** CSA and AGA values across different age groups. Values are expressed as mean ± SD. CSA: critical shoulder angle; AGA: acromioglenoid angle

Age group (years)	CSA (°)	AGA (°)	t-value	p-value
30-39	36.1 ± 2.5	49.2 ± 3.2	26.2	0.01
40-49	35.6 ± 3.0	48.4 ± 3.8	21.1	<0.001
50-59	33.1 ± 2.6	45.4 ± 3.2	24.2	0.05
60-69	33.4 ± 2.9	45.4 ± 3.4	19.2	0.003
70-79	32.8 ± 3.1	44.3 ± 4.3	9.9	<0.001
≥80	31.4 ± 4.9	43.3 ± 3.1	6.1	<0.001

Both CSA and AGA remained highly significant independent predictors of GHOA even after age adjustment (both p < 0.001). Age was a strong independent predictor (OR 1.145 per year, 95% CI 1.098-1.194, p < 0.001), confirming its confounding role as a predictor of GHOA status (Table 3).

**Table 3:** Age-adjusted multivariable logistic regression analysis for predictors of GHOA. CSA: critical shoulder angle; AGA: acromioglenoid angle; GHOA: glenohumeral osteoarthritis; OR: odds ratio; CI: confidence interval

Parameter	OR	95% CI	p-value
CSA (unadjusted)	0.512	0.438-0.597	<0.001
CSA (adjusted)	0.496	0.401-0.613	<0.001
AGA (unadjusted)	0.494	0.421-0.581	<0.001
AGA (adjusted)	0.509	0.417-0.621	<0.001
Age (AGA model)	1.145	1.098-1.194	<0.001

A highly significant association was demonstrated between age group and GHOA status (χ^2^ = 141.5, p < 0.0001). GHOA prevalence was minimal (3.0%) in the 30-39 year age group, progressively increased to 39.0% in the 40-49 age group, and 89.4% in the 50-59 age group, and reached maximum prevalence (95.2% and 100%) in the ≥70-year age groups. This age-stratified analysis demonstrated that advancing age is a significant risk factor for GHOA development in the study population (Table 4).

**Table 4:** Age-stratified distribution of GHOA status showing progressive increase in OA prevalence with advancing age. GHOA: glenohumeral osteoarthritis; OA: osteoarthritis

Age group (years)	GHOA present	Non-OA group	χ^2^ value	p-value
30-39	2	64	141.5	<0.0001
40-49	25	39		
50-59	59	7		
60-69	43	8		
70-79	20	1		
≥80	9	0		

No statistically significant difference in gender distribution was observed between GHOA and non-OA groups (χ^2^ = 0.83, p = 0.36). Males comprised 57.6% of the GHOA group and 52.1% of the non-OA group. Within-gender comparison showed comparable CSA (males: 34.1° ± 3.2° vs. females: 34.8° ± 3.3°, p > 0.05) and AGA (males: 46.6° ± 3.8° vs. females: 47.1° ± 4.1°, p > 0.05) values, indicating no significant gender-specific variation in these radiological parameters (Table 5).

**Table 5:** Gender distribution between GHOA and non-OA groups showing no statistically significant association (p = 0.36). GHOA: glenohumeral osteoarthritis; OA: osteoarthritis

Gender	GHOA present	Non-OA group	Total	χ^2^ value
Male	91	62	153	0.83
Female	67	57	124	
Total	158	119	277	

Excellent inter-observer and intra-observer reliability was demonstrated for both CSA and AGA measurements. For AGA, the inter-observer intraclass correlation coefficient (ICC) was 0.95 (95% CI 0.92-0.97) and the intra-observer ICC was 0.94 (95% CI 0.91-0.96), indicating excellent reliability. Similarly, CSA measurements showed inter-observer ICC of 0.93 (95% CI 0.90-0.95) and intra-observer ICC of 0.92 (95% CI 0.89-0.94).

A significant positive correlation was demonstrated between AGA and CSA in both GHOA and non-OA groups. Among GHOA patients, the correlation coefficient was r = 0.530 (95% CI 0.407-0.634, p < 0.001), indicating a moderate positive association. Among no-OA subjects, the correlation was stronger with r = 0.703 (95% CI 0.599-0.784, p < 0.001), reflecting a strong positive association (Table 6).

**Table 6:** Pearson correlation analysis between the acromioglenoid angle and the critical shoulder angle. CI: confidence interval; GHOA: glenohumeral osteoarthritis; OA: osteoarthritis

Group	n	Correlation coefficient (r)	95% CI	p-value
GHOA group	158	0.530	0.407-0.634	<0.001
Non-OA group	119	0.703	0.599-0.784	<0.001
Overall	277	0.687	0.613-0.754	<0.001

## Discussion

This radiographic investigation shows that there are strong association between both CSA and AGA measures and the development of GHOA. Both parameters are very useful for diagnosis. The average CSA values of 32.5° in GHOA patients and 36.9° in non-OA groups are quite similar to the results of a landmark study by Moor et al., which found average CSA values of 28.1° and 33.1° in OA and control groups, respectively [13]. Our AGA results align with the initial validation study conducted by Miswan et al., which indicated mean AGA values of 45.5° in GHOA patients and 50.9° in controls [15].

The differential correlation strength between CSA and AGA in GHOA versus non-OA groups provides important biochemical insights. The moderate correlation (r = 0.530) in GHOA patients compared to the strong correlation (r = 0.703) in non-OA subjects suggests that osteoarthritic changes alter the normal biomechanical relationship between glenoid inclination and acromial coverage. These findings were consistent with the theoretical model proposed by Moor et al., which depicts that reduced CSA (resulting from inferior glenoid inclination and shortened acromion) increases compressive loading across the shoulder joint, preferentially predisposing to articular cartilage degeneration [13,14]. More recently, Miswan et al.’s prospective analysis demonstrated that AGA and CSA were strongly correlated (r = 0.925, p < 0.001), supporting that both parameters capture related biomechanical variations. However, the authors proposed that AGA is potentially superior due to its reference point at the anatomically significant midglenoid point [15]. Our observation of a slightly larger discriminatory difference with AGA (5.7° vs. 4.4° for CSA) provides support to this argument, though both parameters demonstrated excellent correlation with GHOA status.

Our results confirm and extend findings from multiple prior studies examining CSA in GHOA prediction. Bjarnison et al., in a retrospective case-control study of 87 GHOA patients, demonstrated a 2.25-fold increased risk of OA development with CSA values <30° [19]. Blonna et al. similarly reported that smaller CSA values (28°± 2°) were associated with increased severity of symptomatic OA [18]. Vellingiri et al., in their prospective study on the Indian population, also demonstrate a strong association between GHOA and significantly lower values of CSA (mean = 30.31°) [20]. The reliability of our findings across various study populations and geographic regions reinforces confidence in the validity and generalizability of CSA as a predictive marker.

The gradual decrease in both CSA and AGA values with increasing age found in this study has significant clinical impacts. These results are consistent with the research by Gumina et al., which demonstrates a significant positive linear association between CSA and the increasing age of an individual [21]. This age-related pattern signifies degenerative structural changes involving scapular muscle atrophy, modifications in glenoid morphology, and possible acromial resorption that accumulate over time [22,23]. The gender-independent characteristics of CSA and AGA findings in our study sample (p > 0.05) are significant and align with previous studies [21,24,25]. No significant differences were found regarding the mean CSA and AGA values by side of the shoulder joint.

Biomechanically, a low CSA or AGA implies a steeper glenoid or shorter acromion, which shifts the deltoid vector towards the glenoid surface, which directs the path of degeneration. Clinically, identification of individuals with markedly reduced CSA or AGA values may facilitate early counselling regarding activity modification, strength training protocols, and potentially early intervention in symptomatic patients. In surgical planning, knowledge of CSA and AGA values assists in determining prosthetic choices and alignment parameters during shoulder arthroplasty.

Despite the strong associations, scapular angles are not the sole determinants of OA. Moor et al. noted that individual scapular anatomy is one factor among many [13]. Other factors like genetics, activity level, malalignment, and trauma also play roles [26,27]. Therefore, we do not advocate surgical alteration of scapular anatomy (e.g., acromial trimming) solely based on CSA/AGA, as Bjarnison et al. cautioned against such intervention [19]. However, these measures may aid in risk stratification and guiding conservative management.

Several limitations warrant consideration in interpreting these findings. First, the cross-sectional study design precludes the determination of temporal causality; while our data demonstrate associations between anatomical parameters and GHOA prevalence, prospective longitudinal studies would be required to establish that specific CSA/AGA ranges predict OA development risk in initially symptomatic individuals. Second, convenience sampling from a single tertiary academic center may introduce selection bias, though the demographic characteristics of our cohort generally align with expected epidemiological patterns. Third, radiographic measurements have inherent variability, although we minimized this by using standard views and multiple observers. Future prospective studies should evaluate three-dimensional scapular morphology (e.g., glenoid inclination), which could complement these 2D measures, as suggested by a recent 3D modelling study [28].

## Conclusions

This radiological study found that both the CSA and the AGA are significantly lower in the shoulder with GHOA compared to asymptomatic shoulders. The AGA, a new parameter, showed an even larger mean difference between OA and non-OA groups in our cohort, suggesting it may be at least as useful as CSA for identifying OA-prone scapular morphology. Nonetheless, CSA and AGA are strongly interrelated, and both reflect the underlying scapular geometry that influences joint loading. Clinicians should consider these angles when evaluating shoulder X-rays as a part of a comprehensive assessment of OA risk. Further research is needed to validate these parameters prospectively and to explore their role in clinical decision-making for shoulder degeneration.

## References

[1] Harkness EF, Macfarlane GJ, Silman AJ, McBeth J (2005). Is musculoskeletal pain more common now than 40 years ago?: two population-based cross-sectional studies. Rheumatology (Oxford).

[2] Cadogan A, Laslett M, Hing WA, McNair PJ, Coates MH (2011). A prospective study of shoulder pain in primary care: prevalence of imaged pathology and response to guided diagnostic blocks. BMC Musculoskelet Disord.

[3] Chillemi C, Franceschini V (2013). Shoulder osteoarthritis. Arthritis.

[4] Sambandam SN, Khanna V, Gul A, Mounasamy V (2015). Rotator cuff tears: an evidence based approach. World J Orthop.

[5] Park HB, Gwark JY, Im JH, Jung J, Na JB, Yoon CH (2018). Factors associated with atraumatic posterosuperior rotator cuff tears. J Bone Joint Surg Am.

[6] Yucesoy B, Charles LE, Baker B, Burchfiel CM (2015). Occupational and genetic risk factors for osteoarthritis: a review. Work.

[7] Li X, Olszewski N, Abdul-Rassoul H, Curry EJ, Galvin JW, Eichinger JK (2018). Relationship between the critical shoulder angle and shoulder disease. JBJS Rev.

[8] McLean A, Taylor F (2019). Classifications in brief: Bigliani classification of acromial morphology. Clin Orthop Relat Res.

[9] Aoki M, Ishii S, Usui M, Mizuguchi SM (1972). The slope of the acromion and rotator cuff impingement. Katakansetsu.

[10] Nyffeler RW, Werner CM, Sukthankar A, Schmid MR, Gerber C (2006). Association of a large lateral extension of the acromion with rotator cuff tears. Bone Joint Surg Am.

[11] Hughes RE, Bryant CR, Hall JM (2003). Glenoid inclination is associated with full-thickness rotator cuff tears. Clin Orthop Relat Res.

[12] Tétreault P, Krueger A, Zurakowski D, Gerber C (2004). Glenoid version and rotator cuff tears. J Orthop Res.

[13] Moor BK, Bouaicha S, Rothenfluh DA, Sukthankar A, Gerber C (2013). Is there an association between the individual anatomy of the scapula and the development of rotator cuff tears or osteoarthritis of the glenohumeral joint?: a radiological study of the critical shoulder angle. Bone Joint J.

[14] Viehöfer AF, Snedeker JG, Baumgartner D, Gerber C (2016). Glenohumeral joint reaction forces increase with critical shoulder angles representative of osteoarthritis-a biomechanical analysis. J Orthop Res.

[15] Miswan MF, Saman MS, Hui TS, Al-Fayyadh MZ, Ali MR, Min NW (2017). Correlation between anatomy of the scapula and the incidence of rotator cuff tear and glenohumeral osteoarthritis via radiological study. J Orthop Surg (Hong Kong).

[16] Nwakama AC, Cofield RH, Kavanagh BF, Loehr JF (2000). Semiconstrained total shoulder arthroplasty for glenohumeral arthritis and massive rotator cuff tearing. J Shoulder Elbow Surg.

[17] Standring S (2020). Gray’s Anatomy, 42nd Edition. The Anatomical Basis of Clinical Practice. https://www.scirp.org/reference/referencespapers?referenceid=3108854.

[18] Blonna D, Giani A, Bellato E, Mattei L, Caló M, Rossi R, Castoldi F (2016). Predominance of the critical shoulder angle in the pathogenesis of degenerative diseases of the shoulder. J Shoulder Elbow Surg.

[19] Bjarnison AO, Sørensen TJ, Kallemose T, Barfod KW (2017). The critical shoulder angle is associated with osteoarthritis in the shoulder but not rotator cuff tears: a retrospective case-control study. J Shoulder Elbow Surg.

[20] Vellingiri K, Ethiraj P, Shanthappa AH (2020). Critical shoulder angle and its clinical correlation in shoulder pain. Cureus.

[21] Gumina S, Polizzotti G, Spagnoli A, Carbone S, Candela V (2022). Critical shoulder angle (CSA): age and gender distribution in the general population. J Orthop Traumatol.

[22] Sher JS, Uribe JW, Posada A, Murphy BJ, Zlatkin MB (1995). Abnormal findings on magnetic resonance images of asymptomatic shoulders. J Bone Joint Surg Am.

[23] Nové-Josserand L, Edwards TB, O'Connor DP, Walch G (2005). The acromiohumeral and coracohumeral intervals are abnormal in rotator cuff tears with muscular fatty degeneration. Clin Orthop Relat Res.

[24] Spiegl UJ, Horan MP, Smith SW, Ho CP, Millett PJ (2016). The critical shoulder angle is associated with rotator cuff tears and shoulder osteoarthritis and is better assessed with radiographs over MRI. Knee Surg Sports Traumatol Arthrosc.

[25] Heuberer PR, Plachel F, Willinger L (2017). Critical shoulder angle combined with age predict five shoulder pathologies: a retrospective analysis of 1000 cases. BMC Musculoskelet Disord.

[26] Stamiris D, Stamiris S, Papavasiliou K, Potoupnis M, Tsiridis E, Sarris I (2020). Critical shoulder angle is intrinsically associated with the development of degenerative shoulder diseases: a systematic review. Orthop Rev (Pavia).

[27] Gumina S, Villani C, Carbone S, Venditto T, Candela V (2022). Glenoid version: the role of genetic and environmental factors on its variability. An MRI study on asymptomatic elderly twins. Shoulder Elbow.

[28] Rojas JT, Lädermann A, Dommer L, Jacxsens M, Zumstein MA, Atkins PR (2025). Scapular morphology is associated with certain patterns of glenohumeral osteoarthritis but not with full-thickness rotator cuff tears. J Shoulder Elbow Surg.

